# Longitudinal reciprocal relationship between media violence exposure and aggression among junior high school students in China: a cross-lagged analysis

**DOI:** 10.3389/fpsyg.2024.1441738

**Published:** 2025-01-07

**Authors:** Yifei Dou, Meng Zhang

**Affiliations:** School of Criminology, People’s Public Security University of China, Beijing, China

**Keywords:** media violence exposure, aggression, junior high school students, reciprocal relationship, cross-lagged analysis

## Abstract

**Introduction:**

Increasing evidence has shown that media violence exposure can influence individual aggression. However, the question of whether there is a causal relationship between media violence exposure and aggression remains complex and contentious. This study aims to examine the dynamic reciprocal relations between media violence exposure and aggression among junior high school students in China.

**Methods:**

Using the Exposure to Violent Media Questionnaire (ETVMQ) and the Buss-Warren Aggression questionnaire (BWAQ), 259 junior high school students were tracked three times over a period of 1 year. A cross-lagged panel model was constructed to analyze the reciprocal relationship between media violence exposure and aggression over time.

**Results:**

(1) Media violence exposure and aggression were significantly and positively correlated in all three assessments. (2) Cross-lagged analysis revealed that media violence exposure at Time 1(T1) significantly positively predicted aggression at Time 2(T2), and subsequently significantly positively predicted media violence exposure at Time 3(T3). Additionally, media violence exposure at T2 significantly positively predicted aggression at T3. (3) Multi-group analysis revealed that gender, family economic status, and family location had no significant moderating effects on the cross-lagged effects between media violence exposure and aggression. The cross-lagged effects did not differ by gender, family economic status, or family location.

**Conclusion:**

There is a positive reciprocal relationship between media violence exposure and aggression among Chinese junior high school students, and this reciprocal relationship demonstrates stability across gender and family environments. Media violence exposure is not only a risk factor for increasing aggression among Chinese junior high school students but also a negative outcome of high aggression.

## 1 Introduction

As the internet becomes deeply integrated into the lives and education of adolescents, their opportunities to actively use the Internet are significantly growing, leading to both voluntary and involuntary exposure to vast amounts of violent information based on reality and virtuality (CNNIC, 2023). The connection between the portrayal of media violence and real-life violent scenarios has become increasingly complex, making it challenging to isolate the negative impact of media violence on adolescents from the root. As a special developmental stage, early adolescence becomes a high-risk period for the occurrence of aggressive behavior due to the simultaneous presence of various internal and external changes, such as pubertal development, cognitive transitions, and environmental shifts (Krahé and Busching, 2014; Vitaro and Brendgen, 2012). Contemporary researchers generally divide adolescence into three stages: early adolescence (ages 10–13), middle adolescence (ages 14–17), and late adolescence (ages 18–20) (Smetana et al., 2006; Steinberg, 2007a,b). In China, early adolescence typically corresponds to the junior high school years. Past studies have shown that early adolescents are more susceptible to media violence exposure than late adolescents (Gentile et al., 2014) and behave aggressively (Kirsh, 2003). Many previous studies have focused on early adolescence; for instance, Lin et al. (2022) found a linear increase trend in impulsivity among early adolescents in China, while Ji et al. (2020) reported a co-occurrence of physical and relational aggression among this age group. Kirsh (2006) observed an increase in media consumption from childhood to early adolescence, and he concluded that adolescence appears to be a period during which youths are particularly vulnerable to the effects of media. Compared to other developmental periods, evidence supporting the link between media violence usage and aggression is more consistent during adolescence (Kirsh, 2010). Strengthening the protection of early adolescents on the Internet has become a widespread consensus. Thus, examining the negative impacts of media violence on early adolescents is crucial for reducing their risk of delinquency and fostering their healthy development.

Media violence is generally defined as the depiction or publication of violent content across various media platforms, including movies, television, video games, and newspapers (Li et al., 2019). Media Violence exposure refers to the combined assessment of an individual’s frequency of exposure to violent media and the degree of violence in the content (Gentile et al., 2004). Aggression is defined as any action intended to harm another person, where the aggressor is aware that their actions will harm the target, and the target seeks to avoid such harm (Bushman and Anderson, 2001). Aggression can be categorized into three forms based on the mode of attack: physical aggression, verbal aggression, and indirect aggression (Malti and Rubin, 2018). Previous research has commonly utilized three dimensions—physical aggression, verbal aggression, and indirect aggression—as observation variables to jointly assess the level of aggression in adolescents (Duan et al., 2014; Krahé and Möller, 2010), thereby providing a more comprehensive and accurate depiction of aggression. Specifically, physical aggression, also referred to as direct aggression, includes behaviors such as kicking, hitting, biting, and pushing others forcefully. Verbal aggression involves behaviors such as using profane language, insulting others, mocking or ridiculing, and name-calling. Indirect aggression, also referred to as relational aggression, involves behaviors such as malicious gossip, spreading negative information, inciting physical altercations, and social exclusion (Bushman and Anderson, 2001). It is important to note that there are numerous criteria for classifying aggression based on different research objectives. For example, aggression can be divided into proactive aggression and reactive aggression based on its cause; hostile aggression and instrumental aggression based on its purpose; or implicit aggression and explicit aggression based on cognitive processing mechanisms. In Moyer’s (1968, 1976, 1987) classic works on the psychology of aggression, he identified the following types of aggression: predatory, angry, dominant, sexual, and defensive. Each type serves a specific function through a distinctive emotional state: hunger, anger, competitive drive, sex, and fear. However, based on the classic definition of aggression proposed by Bushman and Anderson (2001) described above and our research objectives, this study categorizes aggression into physical, verbal, and indirect forms for measurement according to different modes of aggression. The types of aggression measured in this study also fall under the categories of explicit aggression and anger aggression (according to Moyer’s classification criteria), which is a response to intentional harm and has been the primary focus of most previous research on aggression. Adolescent aggressive behavior significantly impacts their psychological development and social adaptation (Kawabata and Crick, 2013) and is closely linked to criminal behavior in adulthood. Adolescents exhibiting aggressive behavior are more likely to violate social norms and have a higher risk of criminality (Farrington, 1994). Timely identification and intervention of aggressive behavior in early adolescents are essential for preventing and controlling delinquency in adulthood.

Research on the relationship between media violence and aggression has a long history. Since the advent of television in the last century, psychologists have been exploring the potential connection between exposure to violent media and increased aggression in real life (Eron et al., 1972), and a relatively comprehensive system has been formed. Numerous empirical studies (Anderson et al., 2003; Chen et al., 2012), meta-analyses (Anderson and Bushman, 2001; Anderson et al., 2010; Greitemeyer and Mügge, 2014), and systematic reviews (Teng et al., 2013; Xing et al., 2015) have demonstrated that media violence exposure can influence individual aggression. Watching violent television shows, and movies, and playing violent video games all contribute to an increase in aggressive behavior. However, the question of whether there is a causal relationship between media violence exposure and aggression remains complex and contentious (Ivory, 2013). One perspective posits that media violence is an antecedent variable of aggressive behavior, suggesting a causal relationship, with the General Aggression Model (GAM) proposed by Anderson and Bushman (2002) being a representative theory. The General Aggression Model (GAM) primarily explains the influence of media violence on aggressive behavior through three key components: cognition, emotion, and arousal. It outlines the entire process, including input variables, internal states, and output processes, and distinguishes between short-term effects and long-term effects (Anderson and Bushman, 2002). According to the GAM, input variables consist of individual factors (such as personality traits and physiological attributes) and situational factors (such as media violence). The model posits that individual factors and media violence can influence the evaluation and decision-making process of aggressive behavior through three internal state routes—cognition, emotion, and arousal—and their interactions, ultimately leading to the output of aggressive behavior. Specifically, short-term effects refer to immediate or transient changes in aggressive behavior following brief exposure to media violence, while long-term effects refer to more enduring changes in aggressive behavior resulting from repeated exposure to media violence (Bushman and Huesmann, 2006). Previous research indicates that short-term effects are more obvious in adults, while long-term effects are more obvious in children and adolescents (Bushman and Huesmann, 2006). Moreover, compared with the short-term immediate effects of media violence, its long-term cumulative effects are more worrying (Teng et al., 2019), which can only be measured through longitudinal studies.

Conversely, some scholars argue against this perspective (Ferguson and Kilburn, 2010), proposing the tit-for-tat Catalyst Model (CM), which posits that media violence does not directly affect aggressive behavior but instead acts as a catalyst for aggression induced by other factors such as personality traits and domestic violence (Ferguson et al., 2013). The reason why some studies found a causal relationship between media violence and aggression is because of publication bias, moral panic, and some methodological problems in this research field (Ferguson, 2007). In other words, the catalyst model assumes that media violence exposure is not an antecedent variable to aggressive behavior, but only accelerates or slows down the occurrence of aggressive behavior. If adolescents’ family education, family environment, and physiological factors contain less aggressive factors, then even if they are exposed to media violence, they will have less aggressive behaviors (Ferguson, 2015a). A meta-analysis of 101 studies on the effects of video games on children and adolescents found that video games had very small effects on increased aggressive behavior, reduced academic performance, and depressive symptoms (Ferguson, 2015b). The experimental study by Ferguson et al. (2008) showed that regardless of whether participants were exposed to violent video games or non-violent video games, the difference in aggressive behavior was not significant, but aggressive traits, exposure to domestic violence, and gender were closely related to aggressive behavior. Two other longitudinal studies also found that exposure to video game violence was not associated with any negative outcomes of aggression (Ferguson et al., 2012; Kühn et al., 2019). Some studies have even found that media violence exposure can not only decrease aggressive behavior (Unsworth et al., 2007) but also promote prosocial behavior (Grizzard et al., 2014). It can be seen that both the General Aggression Model and the Catalyst Model have been supported by many empirical studies and meta-analyses, and the debate between the two centers on whether there is a causal link between media violence and aggression. In short, the General Aggression Model believes that there is a causal relationship between media violence and social violence, and media violence is a trigger for criminal behavior. The Catalyst Model believes that the causal relationship between the two is null, and media violence only acts as a stylistic catalyst for criminal behavior. It is not the culprit of various violent behaviors in real society, but just the scapegoat for other social problems. Therefore, the causal relationship between media violence exposure and aggressive behavior remains a topic of intense debate, necessitating more comprehensive and detailed research designs to verify these findings. This also reminds us that, in order to be able to resolve and respond to the debate between the General Aggression Model and the Catalyst Model to a certain extent, the potential influencing factors such as personality traits and family environment factors should be fully taken into account in the research design, and the relationship between media violence exposure and aggressive behavior should be clarified whether the relationship is moderated by personal traits (e.g., gender) and family factors (e.g., family economic status, family location).

The differences in media violence exposure and aggressive behavior across gender and family environments in adolescents have been a common concern for researchers. Numerous studies have shown that boys exhibit higher levels of aggression and more frequent and intense exposure to media violence compared to girls (Gentile et al., 2004; Duan et al., 2014; Zhang et al., 2012). Hawkes (1991) show-off hypothesis also highlights, from an evolutionary perspective, that males display higher levels of aggression than females. Additionally, rural adolescents have been found to engage in higher levels of traditional bullying behaviors, including physical, verbal, and relational aggression, than their urban counterparts (Shen et al., 2019). Adolescents from families with higher socioeconomic status tend to exhibit lower levels of aggression (Tao, 2021). For adolescents in difficult or poor family environments, their parents may need to work outside the home to support the family, resulting in a lack of parental supervision and emotional neglect, which in turn leads to higher levels of media violence exposure and aggression (Yang, 2021). However, this does not necessarily imply that the relationship between media violence exposure and aggression also varies by gender and family environment. In fact, previous research has been inconsistent on this point. A longitudinal study by Huesmann and Miller (1994) found that boys’ preference for television violence at age 8 predicted their aggression at age 18, whereas this was not the case for girls. Anderson and Carnagey (2009) also found that for both males and females who were equally unaccustomed to playing games, males grew significantly more aggressive than females after playing violent games simultaneously. In contrast, Teng et al. (2019) found no significant gender differences in the predictive effect of media violence exposure on aggressive behavior among Chinese adolescents. Some meta-analytic evidence also supports the notion that the relationship between media violence exposure and aggression is similar across gender, age, and family environments (Anderson and Bushman, 2001; Anderson et al., 2010). Therefore, whether gender and family environment moderate the relationship between media violence exposure and aggression remains an issue that requires further investigation.

A review of the relevant literature reveals that most previous theoretical and empirical research has predominantly focused on the unidirectional causal exploration of media violence exposure on aggression (Ybarra et al., 2022; Anderson et al., 2008), mainly through experimental studies examining short-term effects (Prescott et al., 2018). Studies investigating the long-term effects of aggression on media violence exposure are relatively scarce. The relationship between media violence exposure and aggressive behavior may not be a simple unidirectional predictive relationship, and aggressive behavior might also increase an individual’s media violence exposure. However, existing research results are still divided, necessitating further longitudinal studies for clarification. For example, a four-year longitudinal study by Willoughby et al. (2012) found only a positive prediction of violent video games on adolescent aggression, without finding any effect of aggression on violent video games. Similarly, a three-wave longitudinal study by Teng et al. (2019) involving Chinese adolescents confirmed the unidirectional predictive results mentioned above. However, Adachi and Willoughby (2013), through cross-lagged analysis, discovered a bidirectional predictive relationship between competitive video games, competitive gambling, and adolescent aggression over time. In the Social Cognitive Theory (SCT) proposed by Bandura (1989), the model of causality describes the interaction between individual and environmental variables. Bandura (1989) posits that the environment influences individuals through social learning, and conversely, individuals’ behaviors reinforce these environments. Social Cognitive Theory (SCT) indicates that when exposed to violent content in the media environment, early adolescents often engage in indiscriminate observation and imitation, progressively acquiring more complex scripts for aggressive behavior (Bandura, 2001). Through cognitive rehearsal and vicarious reinforcement, their aggressive actions become increasingly refined and readily retrievable from memory, thereby facilitating the development of long-term aggressive behavioral habits. In addition, to explain the causal direction between media violence exposure and aggression, some scholars have proposed two distinct hypotheses: the socialization hypothesis and the selection hypothesis. The socialization hypothesis posits that repeated exposure to media violence and violent social environments causes individuals to become more aggressive (Anderson and Bushman, 2001; Huesmann, 1998). In contrast, the selection hypothesis suggests that individuals who are inherently more aggressive due to biological, genetic, or personality factors tend to seek out violent media content, as it reinforces their belief that their aggressive behaviors are neither uncommon nor unjustified (Huesmann et al., 2003). It is evident that these two hypotheses differ in their starting points, with the former emphasizing environmental influences and the latter focusing on individual predispositions. Further research by Slater et al. (2003) found that both causal pathways can operate simultaneously and mutually reinforce over time, in what they called a downward spiral model. Moreover, the directionality of the relationship between media violence exposure and aggression determines the focus of public policy. If media violence unidirectionally influences juvenile delinquent behavior, then public policy should focus on controlling media content and restricting adolescents’ access to such material. However, if aggressive tendencies can also predict adolescent media violence exposure, then public policy should also consider how to reduce the aggressive personality of adolescents with high crime risk. Based on this, we speculate that media violence as a situational variable may interact with individual aggressive behavior, potentially creating a vicious cycle between the two, in order to provide a more accurate evidence-based basis for the formulation of public policies related to Chinese adolescents.

In addition, most previous studies were conducted in Western countries, such as the United States (Anderson et al., 2008), Canada (Willoughby et al., 2012), Spain (Ferguson et al., 2012), and Germany (Krahé and Möller, 2010). Studies in the context of Eastern culture were mainly concentrated in Japan (Anderson et al., 2008) and Singapore (Gentile et al., 2014). There is still a lack of relevant research on Chinese adolescent groups as subjects, and the publication of relevant papers is concentrated on a few specific Chinese scholars (Teng et al., 2017, 2019, 2020; Xing et al., 2015, 2016; Li et al., 2020). However, the relationship between media violence and aggression may not be a simple academic issue, which involves complex political, economic, and cultural backgrounds (Ferguson, 2015a). Compared with Western individualistic cultures, individuals in Eastern cultures characterized by collectivist values and high moral discipline have lower aggressive behaviors (Bergeron and Schneider, 2005). China also has significant differences from the United States and other Western countries in many known risk factors leading to aggression and violence, such as easy access to firearms. Furthermore, a meta-analysis found that the effect of media violence on aggression was influenced by ethnicity, being greatest among white people, moderate among Asians, and insignificant among Hispanics (Prescott et al., 2018). Even in the era of globalization, the patterns and contexts of media violence exposure and their cultural background may still vary greatly across regions. This reveals that cultural and regional specificities should be fully considered in research, and more relevant empirical research needs to be conducted within the Chinese cultural context to explore the reciprocal relationship between media violence exposure and aggression, thereby providing more targeted empirical evidence for aggression interventions among Chinese adolescents.

In summary, although research exploring the relationship between media violence exposure and aggressive behavior has increased rapidly in recent years, more rigorous and diverse evidence is still needed. First, regarding research design, due to limitations of time and resources, longitudinal studies revealing the dynamic relationship between media violence exposure and aggression over time remain scarce. Cross-sectional studies conducted at a single time point are insufficient to verify causal relationships between variables. Longitudinal designs based on cross-lagged analyses are crucial to examining the causal direction of covariation between media violence exposure and aggression. Furthermore, existing longitudinal studies often have limitations, such as only two measurement points and relatively short time intervals, which weaken the robustness of established causal relationships. To better capture the developmental and dynamic nature of the relationship, studies should include at least three measurement points to explore the reciprocal relationship between the two variables. Second, regarding study populations, prior research has predominantly focused on Western adolescent samples, with insufficient consideration of cultural and regional specificities. Broader data from Chinese populations are needed to test the generalizability of findings and to propose culturally specific intervention strategies tailored to the unique social context and media environment in China. Moreover, from a developmental perspective, early adolescents represent a distinct group whose psychological development and behavioral patterns warrant special attention. Understanding the dynamic reciprocal relationship between media violence exposure and aggression is critical for supporting the growth and adaptation of junior high school students during this key developmental stage. Third, regarding research content, most existing longitudinal studies treat aggressive behavior as the outcome variable of media violence exposure, focusing on the unidirectional predictive mechanism, with limited exploration of reciprocal mechanisms and vicious cycles between the two variables. Additionally, to better address and respond to debates between the General Aggression Model and the Catalyst Model, this study goes beyond identifying the reciprocal relationship between media violence exposure and aggression. It also investigates whether the reciprocal relationship varies by gender and family environment, providing further insights into the robustness of the causal relationship. These findings aim to offer more precise evidence-based recommendations for public policy development.

Therefore, this study aims to employ a three-wave longitudinal design to construct a cross-lagged panel model to deeply explore the dynamic relationship between media violence exposure and aggression among junior high school students in the Chinese cultural context. Furthermore, multi-group analyses will be conducted to assess whether the relationship between media violence exposure and aggression is moderated by adolescents’ gender, family economic status, and family location.

## 2 Materials and methods

### 2.1 Participants and procedures

This study employed a convenience sampling method, selecting students from six classes at a junior high school in China for a three-wave longitudinal study. The initial assessment (T1) included 289 participants, with subsequent assessments conducted at 6-month intervals (T2 and T3). The three waves of survey data were matched and integrated. The questionnaires were screened based on two criteria: whether fixed-choice questions were incorrectly answered (DeSimone et al., 2015) and whether responses demonstrated consistent high-frequency patterns (Curran, 2016). Ultimately, 259 valid participants were retained for the focal variables of this study, resulting in a retention rate of 89.62%. At the time of the first assessment, the participants’ average age was 13.47 years (*SD* = 0.52). Specific demographic variables are shown in Table 1. There were no significant differences between retained and lost participants in terms of gender [*χ^2^*(1) = 0.41, *p* = 0.38], family economic status [*χ^2^*(1) = 0.39, *p* = 0.52], family location [*χ^2^*(1) = 1.12, *p* = 0.43], age [*t*(287) = 1.15, *p* = 0.84], T1 media violence exposure [*t*(287) = −0.51, *p* = 0.65], or T1 aggression [*t*(287) = 0.68, *p* = 0.64], indicating no structural attrition issues.

**Table 1:** Demographic variables.

Variable	Category	Frequency	Percentage
Gender	Male	131	50.58%
	Female	128	49.42%
Family location	Rural	153	59.07%
	Urban	106	40.93%
Family economic status	Higher	118	45.56%
	Lower	141	54.44%

The Criminology Ethics Committee of the People’s Public Security University of China reviewed the study. With informed consent obtained from the class teachers, parents, and students, collective assessments were conducted in classrooms. Trained instructors administered the questionnaires, explaining the purpose of the study and reading the instructions aloud, urging students to respond accurately based on their actual situation. After completing the questionnaires, the instructor collected them and checked for completion. The interval between consecutive assessments was 6 months, and the procedure was consistent across the three waves.

### 2.2 Measures

#### 2.2.1 Media violence exposure

This study utilized the Exposure to Violent Media Questionnaire (ETVMQ), developed by Gentile et al. (2004), which has been validated for reliability and validity in its Chinese version (Duan et al., 2014). The ETVMQ includes two subscales: media exposure frequency and violence preference. Subscale one uses a 5-point Likert scale (1 = once a month or less, 5 = five times a week or more) to assess the frequency of the participant’s usage of three types of media (television, movies/videos, games). Subscale two uses a 5-point Likert scale (1 = no violence, 5 = very violent) to assess the participant’s preference for violent media content. Each subscale consists of three items. The product of the average scores of media usage frequency and violence preference represents the media violence exposure level, with higher scores indicating greater exposure. The Cronbach’s alpha coefficients for this scale in the three assessments were 0.64, 0.71, and 0.78, respectively.

#### 2.2.2 Aggression

Aggression was measured using the Buss-Warren Aggression Questionnaire (BWAQ) developed by Buss and Warren (2000), which has been validated for reliability and validity with Chinese adolescents (Zhang et al., 2011). The questionnaire consists of 19 items, covering three dimensions: physical aggression, verbal aggression, and indirect aggression, rated on a 5-point Likert scale (1 = very uncharacteristic, 5 = very characteristic). Higher scores indicate higher levels of aggression. Furthermore, in order to maintain consistency in the definition and measurement of aggression, we carefully checked each item description in the questionnaire. This process was conducted to ensure that the content and categorization of each item aligned with the research objectives of this study. The Cronbach’s alpha coefficients for this scale in the three assessments were 0.86, 0.87, and 0.88, respectively.

### 2.3 Data analysis

Data were analyzed using SPSS 26.0 and AMOS 26.0. Initially, the questionnaires were inputted, and data from participants who responded irregularly or did not participate in all three assessments were excluded. Subsequently, SPSS 26.0 was used for reliability testing, descriptive statistics, correlation analysis, and common method bias testing. AMOS 26.0 was employed to construct a cross-lagged panel model and perform multi-group analysis, examining model fit using maximum likelihood estimation. In the multi-group analysis, the chi-square difference (Δχ2) between each model was significant (*p* < 0.05) and the model fit indices differences (ΔCFI, ΔTLI, ΔRMSEA) were all >0.01, indicating significant model differences.

## 3 Results

### 3.1 Common method bias test

The Harman single-factor test was used to examine common method bias across all items at the three-time points. The results indicated that there were seven, six, and seven factors with eigenvalues greater than 1 at T1, T2, and T3, respectively. The variance explained by the first factor was 12.47, 15.03, and 12.21% respectively, which were all below the critical threshold of 40% (Podsakoff et al., 2003). This suggests that common method bias is not a significant issue in this study.

### 3.2 Descriptive statistics and correlation analysis

Table 2 presents the means, standard deviations, and Pearson correlations for media violence exposure and the dimensions of aggression across the three-time points. Significant positive correlations were found between media violence exposure and each dimension of aggression at all three-time points, fulfilling the assumptions required for cross-lagged analysis.

**Table 2:** Means, standard deviations, and correlation matrix for media violence exposure and dimensions of aggression across three assessments (*n* = 259).

Variables	*M*	*SD*	1	2	3	4	5	6	7	8	9	10	11	12
1. T1 media violence exposure	5.72	2.82	1											
2. T2 media violence exposure	5.52	2.00	0.52***	1										
3. T3 media violence exposure	6.63	2.92	0.55***	0.63***	1									
4. T1 physical aggression	1.94	0.79	0.46***	0.32***	0.33***	1								
5. T2 physical aggression	1.97	0.79	0.32***	0.30***	0.29***	0.54***	1							
6. T3 physical aggression	2.15	0.90	0.36***	0.32***	0.46***	0.46***	0.53***	1						
7. T1 verbal aggression	2.79	0.80	0.18***	0.19**	0.16**	0.52***	0.27***	0.23***	1					
8. T2 verbal aggression	2.68	0.82	0.15**	0.14*	0.12*	0.34***	0.63***	0.30***	0.36***	1				
9. T3 verbal aggression	2.82	0.85	0.14*	0.21***	0.24***	0.28***	0.34***	0.55***	0.23***	0.42***	1			
10. T1 indirect aggression	2.11	0.76	0.39***	0.32***	0.30***	0.69***	0.26***	0.31***	0.50***	0.32***	0.22***	1		
11. T2 indirect aggression	2.17	0.78	0.19***	0.19***	0.20***	0.39***	0.65***	0.40***	0.30***	0.57***	0.35***	0.39***	1	
12. T3 indirect aggression	2.30	0.81	0.25***	0.20***	0.30***	0.39***	0.42***	0.68***	0.18***	0.30***	0.56***	0.38***	0.51***	1

### 3.3 Cross-lagged analysis of media violence exposure and aggression in junior high school students

To investigate the dynamic reciprocal relationship between media violence exposure and aggression over time and to strengthen causal inference, a cross-lagged panel model was constructed using the data from the three assessments. Media violence exposure was treated as an observed variable, while aggression was modeled as a latent variable comprising verbal aggression, physical aggression, and indirect aggression. The model allowed for correlations between the same variables at different time points and error correlations between variables at the same time point (Little, 2013). Age, gender (0 = male, 1 = female), family economic status (0 = higher, 1 = lower), and family location (0 = rural, 1 = urban) were included as control variables to account for demographic influences. The model’s fit met the ideal criteria set by Hair et al. (2019): χ^2^/df = 2.56(<5), *p* < 0.001, CFI(Comparative Fit Index) = 0.95 (>0.90), TLI(Tucker-Lewis Index) =0.94 (>0.90), RMSEA(Root Mean Square Error of Approximation) = 0.04 (<0.08), SRMR (Standardized Root Mean Square Residual) = 0.08(<0.10).

**Figure 1:**
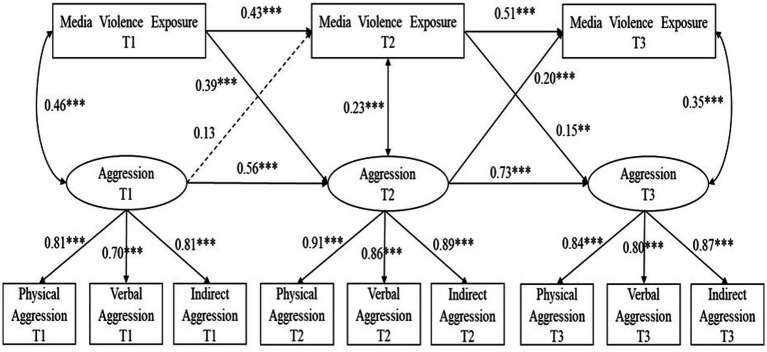
A cross-lagged model of media violence exposure and aggression. *p < 0.05, **p < 0.01, ***p < 0.001. All parameter estimates in the figure are standardized. Solid lines indicate significant effects, while dashed lines indicate non-significant effects. For simplification, the paths of control variables are not shown in the figure.

The cross-lagged path diagram (Figure 1) shows that the autoregressive path coefficients for media violence exposure and aggression across the three-time points ranged from 0.43 to 0.73, indicating a degree of stability. After controlling for the autoregressive effects and the concurrent correlations between media violence exposure and aggression, media violence exposure at T1 significantly positively predicted aggression at T2(*β* = 0.39, *p* < 0.001), and aggression at T2 significantly positively predicted media violence exposure at T3 (*β* = 0.20, *p* < 0.001). The prediction from aggression at T1 to media violence exposure at T2 was not significant (*β* = 0.13, *p* > 0.05), but media violence exposure at T2 significantly positively predicted aggression at T3 (*β* = 0.15, *p* < 0.01).

### 3.4 Multi-group analysis of the cross-lagged model

To further examine whether the cross-lagged effects differ by gender, family economic status, and family location, multi-group analyses were conducted for the cross-lagged model by gender (0 = male, 1 = female), family economic status (0 = higher, 1 = lower), and family location (0 = rural, 1 = urban). Initially, an unconstrained model was established, allowing all paths to be freely estimated. Subsequently, a model with structural weights constrained to be equal across different groups (i.e., gender, family economic status, and family location) was tested and compared with the unconstrained model.

For the gender-constrained model (M2) versus the unconstrained model (M1), the results showed Δχ^2^(8) = 16.41, *p* > 0.05, ΔCFI <0.01, ΔTLI <0.01, ΔRMSEA <0.01. For the family economic status-constrained model (M4) versus the unconstrained model (M3), the results showed Δχ^2^(8) = 11.96, *p* > 0.05, ΔCFI <0.01, ΔTLI <0.01, ΔRMSEA <0.01. For the family location-constrained model (M6) versus the unconstrained model (M5), the results showed Δχ^2^(8) = 16.28, *p* > 0.05, ΔCFI <0.01, ΔTLI <0.01, ΔRMSEA <0.01. Detailed model fit indices are presented in Table 3.

**Table 3:** Model fit indices of multi-group analysis.

Variable	*χ^2^*	*df*	CFI	TLI	RMSEA
Gender					
M1: Unconstrained Model	321.12	122	0.990	0.959	0.061
M2: Gender-Constrained Model	337.53	130	0.982	0.953	0.059
Family economic status					
M3: Unconstrained Model	330.99	122	0.958	0.927	0.070
M4: Family Economic Status-Constrained Model	342.95	130	0.956	0.919	0.069
Family location					
M5: Unconstrained Model	359.92	122	0.931	0.896	0.078
M6: Family Location-Constrained Model	376.20	130	0.928	0.894	0.076

These results indicated that constraining path equality did not significantly affect the model. Overall, based on the differences in various indices, the cross-lagged effects between media violence exposure and aggression did not significantly vary among adolescents of different genders, family economic statuses, or family locations in this study. In other words, gender, family economic status, and family location had no significant moderating effects on the cross-lagged effect between media violence exposure and aggression, suggesting that the cross-lagged model, as shown in Figure 1, exhibits strong robustness.

## 4 Discussion

Based on the General Aggression Model (GAM) and Social Cognitive Theory (SCT), this study investigated the reciprocal relationship between media violence exposure and aggression among Chinese junior high school students through a one-year, three-wave longitudinal study employing a cross-lagged panel design. Correlation analyses revealed significant positive correlations between media violence exposure and physical aggression, verbal aggression, and indirect aggression, indicating that higher levels of media violence exposure were associated with higher levels of these forms of aggression. These findings are consistent with previous cross-sectional studies conducted within the Chinese cultural context (Zhang et al., 2012; Duan et al., 2014). Building on the significant positive correlations between media violence exposure and aggression, this study further explored their reciprocal relationship using a three-wave cross-lagged panel design. The results demonstrated that media violence exposure at Time 1 (T1) significantly positively predicted aggression at Time 2 (T2), which subsequently significantly positively predicted media violence exposure at Time 3 (T3). Furthermore, media violence exposure at T2 significantly positively predicted aggression at T3. These findings validate the hypothesis of a positive reciprocal relationship between media violence exposure and aggression among junior high school students, consistent with longitudinal findings by Adachi and Willoughby (2013) and supporting Bandura’s (2001) Social Cognitive Theory, which posits that media violence, as an environmental variable, interacts with aggression, an individual variable.

The finding that media violence exposure can positively predict aggression six months later supports the validity of the General Aggression Model (Anderson and Bushman, 2002) and partially addresses the debate regarding their causal relationship (Anderson and Bushman, 2001; Ferguson et al., 2013). This result suggests that media violence exposure has both immediate and sustained cross-temporal effects on early adolescents’ aggressive tendencies and behaviors. Bushman (2016) found that the effects of media violence exposure accumulated over time. Huesmann et al. (2003) also found in a 15-year longitudinal study that children who were heavily exposed to television violence in elementary school tended to display higher levels of aggression as teenagers and were more likely to be arrested and prosecuted for criminal behavior in adulthood. As an environmental variable, media violence affects users’ internal states by altering their aggressive cognitions and emotions (Krahé et al., 2011), desensitizing them emotionally to violent scenes, increasing their tolerance for violence, and shaping aggression schemas in which violence is viewed as a reasonable means to solve problems (Anderson et al., 2010), which leads to more frequent aggressive behavior. Based on the General Aggression Model (Anderson and Bushman, 2002), anger, an important aggressive emotion, may play a critical role in the process by which media violence exposure influences aggression. Anger is generally considered an emotional preparation factor for aggressive behavior, as it activates aggressive scripts and patterns composed of hostile thoughts and impulsive tendencies when an individual experiences anger (Berkowitz, 1990). In such states, attention is directed toward anger-related information (Eckhardt and Cohen, 1997), which influences one’s interpretation of situations and greatly increases the likelihood of engaging in aggressive behavior. The role of anger becomes even more pronounced when media violence involves clear provocation or frustration (Han et al., 2020). This effect is especially likely to manifest in early adolescents with poor executive control ability, as they find it particularly difficult to inhibit anger-related behaviors and shift attention away from anger-inducing thoughts (Denson et al., 2012). This finding suggests that future studies could explore the longitudinal mediating roles of cognitive variables such as aggressive normative beliefs (Duan et al., 2014) and moral disengagement (Teng et al., 2017), as well as emotional variables such as empathy (Duan et al., 2014) and anger(Han et al., 2020) between media violence exposure and aggression.

Interestingly, we also found that aggression at T2 significantly positively predicted media violence exposure at T3. Junior high school students, being in adolescence, have strong impulses and a desire for adventure (Steinberg, 2007a,b) and are often unable to resist external temptations. Frequent aggressive behaviors are a notable characteristic of adolescence (Huesmann and Eron, 1986). Considering media violence as a form of adventurous behavior, more aggressive junior high school students may be more likely to seek out adventurous activities, including media violence, out of curiosity (Adachi and Willoughby, 2013). Higher levels of aggression may indicate the formation of aggression schemas (Bandura, 2001; Anderson and Huesmann, 2003), with frequently activated aggression schemas leading to the automatization and habitualization of aggression-related thoughts, emotions, and behaviors, even potentially becoming part of one’s personality traits (Bartholow et al., 2005). Consequently, these individuals may actively seek out violent media for psychological pleasure and satisfaction. Moreover, to increase user engagement, media platforms may use algorithms to filter and recommend content based on user preferences and feedback (Willson, 2017), leading highly aggressive individuals to encounter violent media more frequently and deeply, reinforcing their violent biases and trapping them in an information cocoon of aggression (Sunstein, 2006), thereby creating a vicious cycle.

It is worth noting that, in addition to identifying the chain reaction and reciprocal effects of the relationship between media violence exposure and aggression in this study (T1 media violence exposure → T2 aggression → T3 media violence exposure), the results of the cross-lagged analysis also indicated that T1 aggression did not significantly predict T2 media violence exposure. The result of this pathway does not support the selection hypothesis (Huesmann et al., 2003) and to some extent suggests that media violence exposure, as an environmental factor, plays an important triggering role in the chain of aggression formation. Even if individuals initially possess higher biological and genetic predispositions related to aggression, they may not actively seek violent content or engage in externalized aggressive behavior without exposure to high-risk environmental factors such as media violence. The reciprocal relationship described above only seems to be initiated once media violence exposure occurs. Although previous research has shown that aggression is highly heritable (Fernàndez-Castillo and Cormand, 2016), the biological mechanisms and the interplay between genes and environment that result in aggression remain elusive (Koyama et al., 2024). Craig and Halton's (2009) study found that the genetic effects on the aggressive phenotype depend upon environmental factors such as family adversity, antisocial disadvantage, violent media exposure, or alcohol use. Additionally, a review by Tuvblad and Baker (2011) indicated that the development of aggression is influenced by gene–environment interaction (G × E) and that genetic predispositions may have different effects depending on the environment. These findings demonstrate the significant role of media violence exposure as an environmental factor in the development of individual aggression. Of course, we are neither slaves to the genes nor the environment. In exploring the causes of aggression, it is essential to adopt a dynamic perspective, fully recognizing the contributions of both individual and environmental factors. A developmental perspective of aggression is based on the assumption that aggressive behavior is multidetermined and dynamic over the lifespan and is a product of a complex continuous interaction of multiple psycho-bio-social changes (Ramirez, 2003). For example, a healthy and civilized online environment, a positive and friendly interpersonal climate, and effective policing may reduce violence levels among adolescents with antisocial tendencies. Conversely, adolescents exposed to media violence or those who have experienced neglect or abuse are more likely to exhibit aggressive behaviors. This provides further support for focusing on the plasticity and developmental nature of adolescent psychology and behavior from an environmental perspective.

Furthermore, to further address the debate between the General Aggression Model and the Catalyst Model, this study thoroughly considered the influence of personality traits and family environment factors among junior high school students. Through multi-group analysis, it was found that gender, family economic status, and family location of junior high school students did not moderate the cross-lagged effects between media violence exposure and aggression. This finding suggests that the positive reciprocal relationship between media violence exposure and aggression is robust, showing little influence from gender and family environment factors. It is not difficult to see that this result is completely opposite to the perspective of the Catalyst Model. According to the Catalyst Model (Ferguson et al., 2012), the effects of media violence on aggressive behavior should exhibit significant individual differences. Individuals with aggressive traits or those exposed to family violence are expected to show accelerated development of aggressive behaviors after being exposed to media violence. Conversely, if the family environment and physiological factors of adolescents contain fewer aggressive factors, then even if they are exposed to media violence, they will have less aggressive behavior (Ferguson et al., 2008). However, our study consistently found that media violence exposure had a positive predictive effect on aggression across different genders, family economic status, and family location groups, indicating that this predictive effect is consistent across genders and family environments. This finding refutes the Catalyst Model and provides strong support for the General Aggression Model, reinforcing the notion that media violence exposure is indeed an antecedent variable to aggressive behavior.

Although many previous studies have demonstrated gender and family environment differences at the individual level of each variable of media violence exposure and aggression, the present study suggests that no such differences are evident in the relationship between media violence exposure and aggression. This finding is consistent with prior research conducted with Chinese adolescents (Teng et al., 2019) and relevant meta-analyses (Anderson and Bushman, 2001; Anderson et al., 2010). The underlying reasons for this result may be related to the ubiquity and accessibility of internet media usage in contemporary China. Whether boys or girls, growing up in rural or urban areas, with higher or lower family economic status, the widespread use of internet media has ushered Chinese adolescents into an era of explosive presentation of violent content (CNNIC, 2023). As adolescents repeatedly encounter violent information or experiences in the media, they can easily develop aggressive cognitive schema, which may inevitably trigger aggressive behavior (Krahé et al., 2011). Vice versa, television, videos, and games have become almost universal among contemporary Chinese adolescents. When aggressive behavior occurs, coupled with the fact that personal aggression schemas may have already been formed, it may be a common practice for today’s adolescents to obtain psychological satisfaction and comfort by browsing relevant information and scenarios in the media (Adachi and Willoughby, 2013). This result also suggests that schools and society should treat students equally and adopt uniform measures when preventing and intervening in media violence and aggressive behavior among adolescents, avoiding biases or special treatment towards specific groups. Future research should include more family-related factors (e.g., parental marital status, only-child status, and left-behind children status) to further explore their moderating effects on the relationship between media violence exposure and aggression, thereby providing targeted intervention strategies for relevant populations.

Overall, the primary finding of this study is that media violence exposure at earlier assessments (T_n_) can significantly positively predict aggression at subsequent assessments (T_n + 1_), which in turn can significantly positively predict media violence exposure at later assessments (T_n + 2_). Moreover, this finding demonstrates stability across different genders and family environments. This reveals a reciprocal causality between media violence exposure and aggression among Chinese junior high school students, where media violence exposure is both a long-term risk factor for increasing aggression and a negative outcome of high levels of aggression. This finding has important implications for the prevention and intervention of aggressive behavior in junior high school students. Governments, internet platforms, schools, and families must work collaboratively, addressing both environmental and individual factors while balancing media environment purification with adolescent education. On the one hand, external exposure to media violence among junior high school students should be controlled and reduced by limiting the content and duration of their use of media devices such as smartphones, computers, and televisions. On the other hand, adolescents’ aggressive behaviors should be corrected promptly by reshaping their cognitive aggression schemas and guiding them to learn from positive role models, thereby breaking the vicious cycle between media violence exposure and aggression and fostering a virtuous cycle. Moreover, given the significant triggering role of media violence exposure as an environmental factor in the causal chain of aggression formation, particular emphasis should be placed on creating a safe and healthy media environment for adolescents. This can be achieved through a multi-pronged approach, including identity verification, time management, spending limits, content filtering, violence classification, and internet education, which aim to reduce the risk factors associated with media violence that contribute to juvenile delinquency.

## 5 Limitations and recommendations

However, this study has several limitations that need to be addressed in future research.

First, although this study conducted a one-year, three-wave longitudinal study, revealing causal relationships between media violence exposure and aggression, the tracking duration is still relatively short, and the number of tracking points needs to be increased. Future research could extend the tracking period and increase the number of tracking points, utilizing longitudinal analysis methods such as latent transition analysis and latent mixture growth modeling, to more accurately examine the dynamic trends of media violence and aggressive behavior throughout the entire developmental stage of adolescents.

Second, the sample size of this study was relatively small and was only selected from one junior high school. Although the sample distribution across demographic variables was relatively balanced (see Table 1) and demographic variables were controlled for during data analysis, differences in educational quality and management styles among schools in Chinese provinces with varying levels of economic development may influence students’ media violence exposure and aggressive behaviors. This limitation reduces the stability and representativeness of the findings to some extent. Caution should be taken when generalizing these results to other regions and populations. Future studies could conduct regional stratified sampling across China, covering regions with different levels of economic development, to obtain larger and more representative samples. Additionally, other groups such as children, college students, and adults could be included to test the universality of the reciprocal mechanism between media violence exposure and aggression. Moreover, this study focuses on media violence exposure and aggressive behavior among Chinese adolescents. Even in the era of globalization, patterns of media violence exposure and the cultural contexts in which they occur may vary significantly across countries. Future research could compare data from China with that of other regions and countries, such as Southeast Asia, Europe, and the United States, to examine the generalizability of these findings using a broader dataset from across the globe.

Third, this study used self-report questionnaires for all variables, which are subject to inherent subjective bias. The use of questionnaires also constrained the investigation to the long-term effects of media violence exposure on aggression, neglecting potential short-term effects. There are obvious differences in the psychological processes of short-term and long-term effects (Bushman and Huesmann, 2006). Methodologically, correlational studies are typically used to explore long-term effects, while experimental studies are more suited for examining short-term effects (Anderson and Bushman, 2001; Wei et al., 2010). Therefore, it is necessary to distinguish the two effects of media violence in future studies. Previous research has shown that media violence exposure can be experimentally manipulated by engaging in violent games or watching violent videos (Guo et al., 2009), and aggression includes both explicit and implicit aggression, each with different measurement paradigms (Yang and Liu, 1996). Future research should consider combining self-report questionnaires with peer-report questionnaires, situational experiments, and implicit association tests to obtain more objective and diverse data sources, distinguish the long-term and short-term effects of media violence on adolescent aggression, thus providing a more comprehensive examination of the effects and mechanisms between media violence exposure and aggression.

Fourth, this study only focused on the reciprocal predictive relationship between media violence exposure and aggression and explored the moderating roles of three demographic variables such as gender, family economic status, and family location through multiple-group analyses. However, the exploration of potential mediating and moderating variables was insufficient. Based on the General Aggression Model, there may be many mediators between media violence exposure and aggression in the three aspects of cognition, emotion, and arousal, including cognitive variables such as aggressive beliefs (Duan et al., 2014) and moral disengagement (Teng et al., 2017), emotional variables such as anger (Han et al., 2020) and empathy (Duan et al., 2014), and arousal variables such as skin conductance (Ivory and Kalyanaraman, 2007) and heart rate (Barlett et al., 2008). Similarly, in terms of the moderating mechanisms, this study only examined the moderating effects of three individual-level demographic variables, leaving room for the exploration of other potential moderators, such as trait anger (Li, 2022), trait empathy (Krahé and Möller, 2010), and trait aggression (Teng et al., 2020). Future research should continue to use longitudinal studies to further explore the complex mechanisms underlying the relationship between media violence exposure and aggression, providing solid empirical evidence for theoretical advancements.

## 6 Conclusion

The following conclusions were obtained from this study: (1) Media violence exposure and aggression were significantly and positively correlated in all three assessments. (2) Media violence exposure at Time 1(T1) significantly positively predicted aggression at Time 2(T2), which subsequently significantly positively predicted media violence exposure at Time 3(T3). However, aggression at T1 did not significantly predict media violence exposure at T2, but media violence exposure at T2 significantly positively predicted aggression at T3. (3) Gender, family economic status, and family location had no significant moderating effects on the cross-lagged effects between media violence exposure and aggression. The cross-lagged effects did not differ by gender, family economic status, or family location. These findings indicate a positive reciprocal relationship between media violence exposure and aggression among junior high school students in China, and this reciprocal relationship demonstrates stability across gender and family environments. Media violence exposure is not only a risk factor for increasing aggression but also a negative outcome of high aggression.

## Data Availability

The original contributions presented in the study are included in the article/supplementary material, further inquiries can be directed to the corresponding author.
